# Utilizing serum metabolomics for assessing postoperative efficacy and monitoring recurrence in gastric cancer patients

**DOI:** 10.1186/s12885-023-11786-2

**Published:** 2024-01-02

**Authors:** Tong Qu, Shaopeng Zhang, Shaokang Yang, Shuang Li, Daguang Wang

**Affiliations:** Department of Gastrocolorectal Surgery, General Surgery Center, The First Hospital of Jilin University, 71 Xinmin Street, 130021 Changchun, Jilin P.R. China

**Keywords:** Gastric cancer, Serum metabolites, Postoperative efficacy, Recurrence monitoring, Biomarkers

## Abstract

(1) This study aims to identify distinct serum metabolites in gastric cancer patients compared to healthy individuals, providing valuable insights into postoperative efficacy evaluation and monitoring of gastric cancer recurrence; (2) Methods: Serum samples were collected from 15 healthy individuals, 16 gastric cancer patients before surgery, 3 months after surgery, 6 months after surgery, and 15 gastric cancer recurrence patients. T-test and analysis of variance (ANOVA) were performed to screen 489 differential metabolites between the preoperative group and the healthy control group. Based on the level of the above metabolites in the recurrence, preoperative, three-month postoperative, and six-month postoperative groups, we further selected 18 significant differential metabolites by ANOVA and partial least squares discriminant analysis (PLS-DA). The result of hierarchical clustering analysis about the above metabolites showed that the samples were regrouped into the tumor-bearing group (comprising the original recurrence and preoperative groups) and the tumor-free group (comprising the original three-month postoperative and six-month postoperative groups). Based on the results of PLS-DA, 7 differential metabolites (VIP > 1.0) were further selected to distinguish the tumor-bearing group and the tumor-free group. Finally, the results of hierarchical clustering analysis showed that these 7 metabolites could well identify gastric cancer recurrence; (3) Results: Lysophosphatidic acids, triglycerides, lysine, and sphingosine-1-phosphate were significantly elevated in the three-month postoperative, six-month postoperative, and healthy control groups, compared to the preoperative and recurrence groups. Conversely, phosphatidylcholine, oxidized ceramide, and phosphatidylglycerol were significantly reduced in the three-month postoperative, six-month postoperative, and healthy control groups compared to the preoperative and recurrence groups. However, these substances did not show significant differences between the preoperative and recurrence groups, nor between the three-month postoperative, six-month postoperative, and healthy control groups; (4) Conclusions: Our findings demonstrate the presence of distinct metabolites in the serum of gastric cancer patients compared to healthy individuals. Lysophosphatidic acid, triglycerides, lysine, sphingosine-1-phosphate, phosphatidylcholine, oxidized ceramide, and phosphatidylglycerol hold potential as biomarkers for evaluating postoperative efficacy and monitoring recurrence in gastric cancer patients. These metabolites exhibit varying concentrations across different sample categories.

**Supplementary Information:**

The online version contains supplementary material available at 10.1186/s12885-023-11786-2.

## Introduction

Gastric cancer is a major global health concern, with more than one million newly diagnosed cases yearly and approximately 800,000 deaths in 2020 [1]. Despite declining cancer incidence and mortality rates worldwide over the past five decades, gastric cancer remains the third leading cause of cancer-related deaths [2, 3]. Notably, epidemiological studies have revealed an alarming rise in gastric cancer incidence among young individuals, potentially linked to factors such as autoimmune responses [4, 5]. Consequently, gastric cancer has become a critical subject of research. Surgical resection has demonstrated improved survival rates for early-stage gastric cancer compared to endoscopic resection [6], while for advanced-stage cases, surgical resection remains the sole curative treatment option. Nevertheless, despite advancements in treatment outcomes [7–9], the overall survival rate for recurrent gastric cancer patients remains low [10]. Early detection of postoperative recurrence plays a crucial role in enhancing patient prognosis. Current clinical methods for recurrence monitoring include abdominal CT, endoscopic biopsy, and serum tumor markers. However, these methods have limitations in detecting small recurrent lesions and may cause discomfort. Therefore, identifying specific biomarkers for assessing and predicting gastric cancer occurrence, metastasis, and treatment response is paramount. Metabolomics, a post-genomic research field, holds immense potential in unraveling complex disease mechanisms by analyzing low-molecular-weight compounds in biological samples. It has played a vital role in diagnosing various cancers and other diseases, making it an ideal tool for this research [11]. In this study, we focused on investigating serum metabolites in gastric cancer patients, revealing significant increases in phosphatidylcholine, oxidized ceramide, and phosphatidylglycerol levels, while Lysophosphatidic acid, triglycerides, lysine, and Sphingosine-1-phosphate were significantly decreased [12, 13]. By shedding light on these differential serum metabolites, our research aims to contribute to evaluating gastric cancer surgical efficacy and postoperative recurrence monitoring, with the ultimate goal of improving patient outcomes.

## Materials and methods

### Patients

The study received approval from the Ethics Committee of The First Hospital of Jilin University (Changchun, China), and written informed consent was obtained from all patients. Serum samples were collected from pathologically confirmed gastric cancer patients who received treatment at the Department of Gastrointestinal and Colorectal Surgery, The First Hospital of Jilin University, between June 2021 and January 2022. We collected serum samples from 15 healthy individuals and 16 gastric cancer patients before surgery, 3 months and 6 months after surgery. Inclusion criteria for these 16 patients: (1) Patients with a single primary tumor without distant metastasis; (2) No history of diabetes or other major diseases and good cardiac and hepatic-renal function; (3) Patients not receiving relevant treatments; (4) Absence of evident acute inflammatory diseases. Exclusion criteria for these 16 patients: (1) Patients with impaired hepatic-renal-cardiac-pulmonary function; (2) Patients with acute inflammatory disease or major stress response in the past two weeks; (3) Patients with metabolic diseases (e.g., diabetes) or hematologic diseases; (4) Patients with infectious diseases (e.g., hepatitis B); (5) Pregnant or lactating women; (6) Individuals with substance abuse, drug addiction, or prolonged use of steroids. In addition, we collected serum samples from 15 patients with recurrent gastric cancer. Recurrence criteria: (1) Histologically confirmed presence of cancer cells at the anastomosis site via endoscopic biopsy; (2) Evidence of liver metastasis on contrast-enhanced abdominal CT; (3) Cancer cells detected in peritoneal effusion cell examination; (4) Mesenteric lymph node metastasis indicated by PET-CT.

### Sample collection

Venous blood samples were collected in the early morning from fasting subjects and centrifuged at 3000 rpm for 10 min at 4 °C. The upper serum was extracted and stored at -80 °C until further use. For analysis, 100 µL of plasma was precisely aspirated into a 1.5 ml EP tube and mixed with a 4-fold volume of methanol: acetonitrile (1:1, V/V). The mixture was vortexed for 30 s, sonicated in an ice bath for 10 min, and kept in a -20 °C refrigerator for 1 h. Afterward, the mixture was centrifuged at 13,500 rpm for 10 min at 4 °C, and the supernatant was collected for injection analysis.

### LC-MS

LC-MS analysis was performed using the AB Sciex Triple TOF 5600TM system (AB Sciex, America) and the Exion UHPLC system (Shimadzu, Japan). Sample mixing was carried out using Vortex 3 from Germany (IKA), and centrifugation was conducted using the H165R low-temperature centrifuge (4℃) from Xiangyi Centrifuge Instrument Co., Ltd. (China). The solvents used were acetonitrile (HPLC grade, Sigma-Aldrich, America), formic acid (HPLC grade, Sigma-Aldrich, America), methanol (HPLC grade, Merk, Germany), and deionized water (Watson, China). Positive and negative ion calibration solutions (POS and NEG, respectively) from AB Sciex were used for ion calibration. The liquid chromatography system employed was the Exion UHPLC System with a binary gradient pump. The chromatographic column was the ACQUITY UPLC HSS T3 (2.1 × 100 mm, 1.8 μm, Waters, USA). The mobile phase comprised solvent A (0.1% formic acid in water) and solvent B (pure acetonitrile). The column temperature was set at 35℃. The injection volume for both positive (POS) and negative (NEG) modes was 5 µL. The elution program was carried out with a linear gradient. Before the formal sample analysis, a 5-minute equilibration with the initial mobile phase was performed to ensure the stability of the liquid phase system and chromatographic column. Ten injections of quality control (QC) samples were used to ensure the accuracy and reliability of the experimental data. The flow rate was maintained at 0.35 ml/min. The gradient time was set as follows: at 0.5 min, A phase was 98%, and B phase was 2%; at 1.5 min, A phase was 80%, and B phase was 20%; at 4 min, A phase was 35%, and B phase was 65%; at 11 min, A phase was 5%, and B phase was 95%; at 15 min, A phase was 5%, and B phase was 95%; at 15.1 min, A phase was 98%, and B phase was 2%; at 20 min, A phase was 98%, and B phase was 2%.

### Mass spectrometry

Mass spectrometry analysis was performed using the AB Sciex TripleTOF 5600 system in both positive and negative ion modes with an electrospray ionization source. The first-level mass spectrometry parameters for the ion source were as follows: in positive mode, ion spray voltage (V) was set to 5500; temperature (℃) to 550; gas 1 (psi) to 55; gas 2 (psi) to 55; curtain gas (psi) to 30; declustering potential (DP) to 100; collision energy (CE) to 10; in negative mode, ion spray voltage (V) was − 4500; temperature (℃) to 550; gas 1 (psi) to 55; gas 2 (psi) to 55; curtain gas (psi) to 30; declustering potential (DP) to -100; collision energy (CE) to -10. The second-level mass spectrometry parameters for the ion source were as follows: in positive mode, ion spray voltage (V) was set to 5500; temperature (℃) to 550; gas 1 (psi) to 55; gas 2 (psi) to 55; curtain gas (psi) to 30; declustering potential (DP) to 100; collision energy (CE) to 35; collision energy spread (CES) to 15; ion release delay (IRD) to 67; ion release width (IRW) to 25; in negative mode, ion spray voltage (V) was − 4500; temperature (℃) to 550; gas 1 (psi) to 55; gas 2 (psi) to 55; curtain gas (psi) to 30; declustering potential (DP) to -100; collision energy (CE) to -35; collision energy spread (CES) to 15; ion release delay (IRD) to 67; ion release width (IRW) to 25.

### Statistical analysis

The LC-MS data were collected using the Analyst TF1.7.1 software (AB Sciex) and processed with PeakView2.2 (AB Sciex). The special format raw data files were imported into the XCMS software for relevant preprocessing. MetaboAnalyst 5.0 was utilized for principal component analysis (PCA) and T-test (P < 0.01) to initially screen the different metabolites between the preoperative and the healthy control groups. Subsequently, ANOVA (P < 0.01) and PLS-DA (with variable importance in projection, VIP > 1.2) were performed to further select different metabolites. Next, the samples were re-grouped into the tumor-bearing group (preoperative and recurrence groups) and the tumor-free group (three-month postoperative and six-month postoperative groups) using clustering analysis. A further PLS-DA (with variable importance in projection, VIP > 1.0) was performed on the re-grouped samples to identify different metabolites. To find metabolites with good discriminatory ability among the sample groups, ROC curves were drawn using the SPSS Statistics 21 software. The selected metabolites were subjected to hierarchical clustering analysis in the healthy control group, preoperative group, recurrence group, three-month postoperative group, and six-month postoperative group. Mean plots were generated using GraphPad Prism to observe the patterns of change in the different metabolites. Finally, the identified metabolites were cross-referenced with the HMDB to determine their substance structures and names.

## Results

### Identification of differential metabolites and distinct sample Ggroup separation in LC-MS analysis

**Fig. 1:**
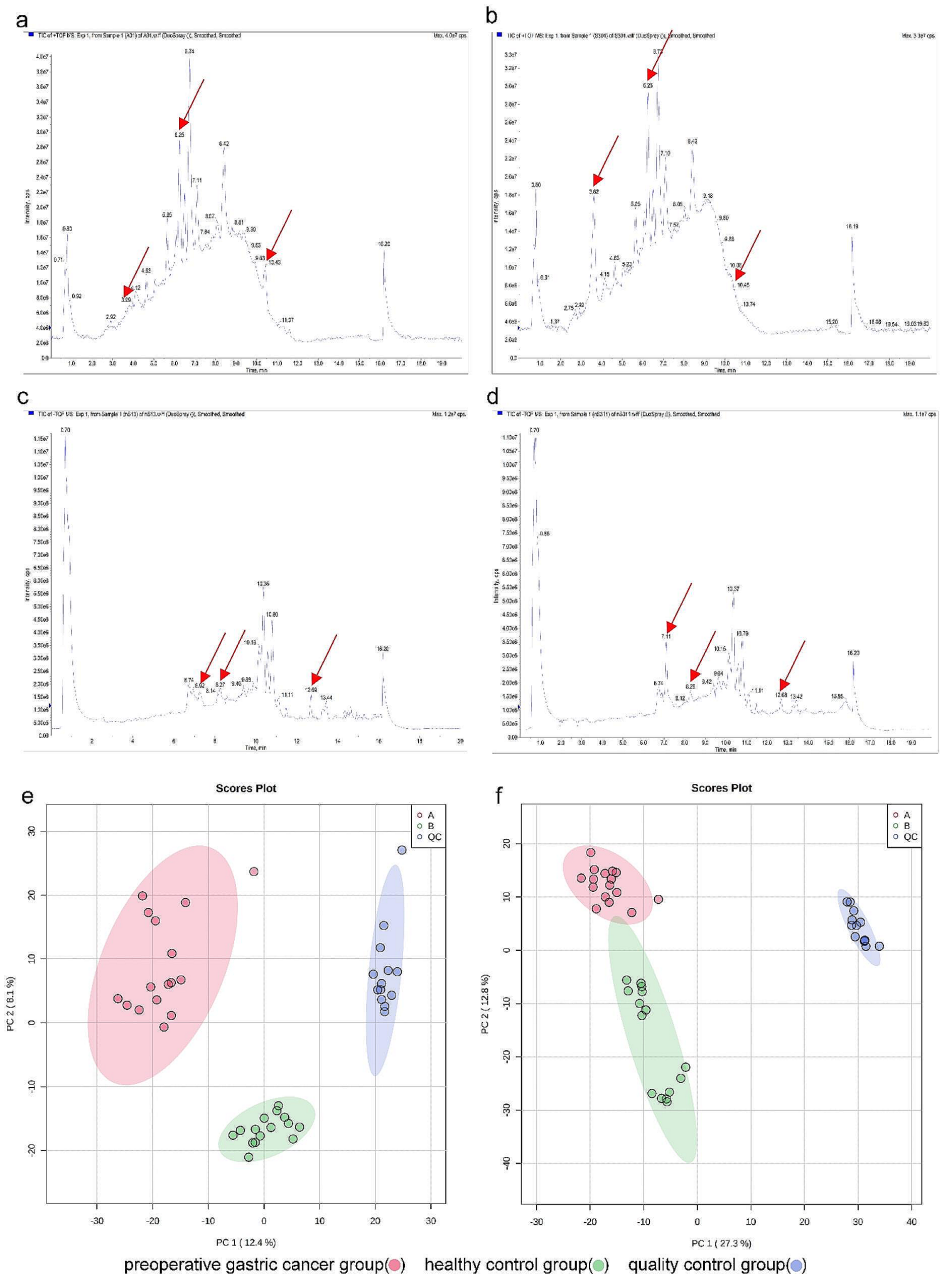
Visualization of Total Ion Chromatograms and PCA Plots in Positive and Negative Ion Modes. (**a**) The total ion chromatogram of the preoperative group in positive ion mode; (**b**) The total ion chromatogram of the healthy control group in positive ion mode; (**c**) The total ion chromatogram of the preoperative group in negative ion mode; (**d**) the total ion chromatogram of the healthy control group in negative ion mode; (**e**) The PCA plot in positive ion mode; (**f**) The PCA plot in negative ion mode

The total ion chromatograms of the preoperative group (Fig. 1a, 1c) and the healthy control group (Fig. 1b, 1d) were analyzed. The results revealed significant differences in peak intensities of specific metabolites between the preoperative and the healthy control groups at the same retention time and different ion modes, indicating the presence of distinct metabolites between the two groups.

Unsupervised PCA was conducted to analyze the samples from the preoperative gastric cancer, healthy control, and quality control (QC) groups (Fig. 1e, 1f). The findings demonstrated clear separations between the three groups in both positive and negative ion modes. Samples with pronounced differences in metabolites exhibited more considerable distances between them, while samples with minor differences in metabolites showed closer distances. The QC group, which represented a mixture of all samples, exhibited consistent metabolite profiles, confirming the reliability of the experimental results.

### Metabolic differences between the preoperative gastric cancer and healthy control groups using T-test and variance analysis

**Fig. 2:**
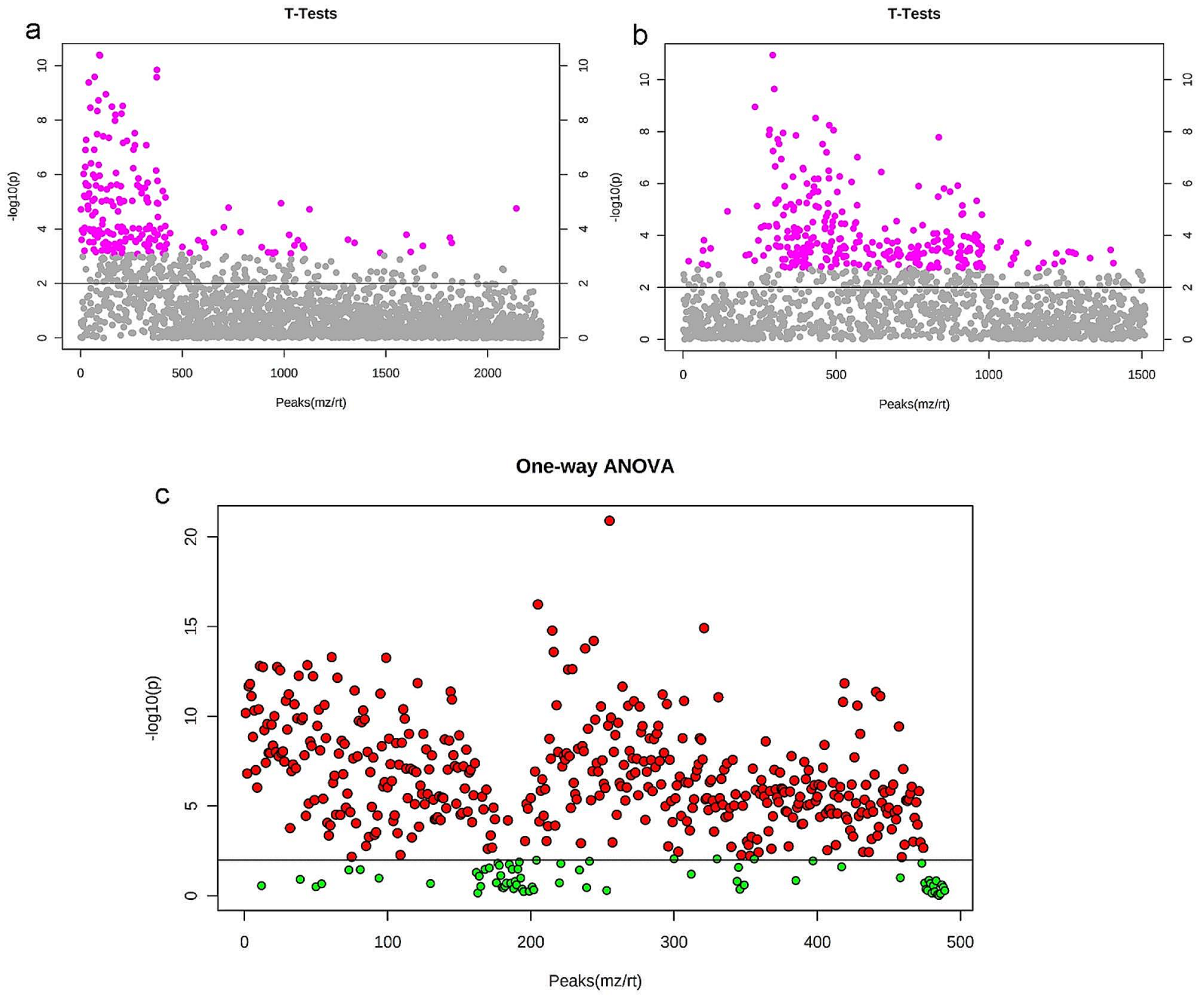
Comparative T-test and Variance Analysis Plots in Positive and Negative Ion Modes. (**a**) The T-test analysis plot in positive ion mode; (**b**) T-test analysis plot in negative ion mode. The y-axis represents the magnitude of P-values, where larger values indicate smaller P-values, and the x-axis represents the relative abundance of each metabolite. In the plot, the purple area represents the metabolites with P-values < 0.01; (**c**) The variance analysis plot of differential metabolites. The red area in the plot represents the metabolites with P-values < 0.01

Under different modes, gastric cancer and the healthy control groups underwent T-test analysis (Fig. 2a, 2b). Metabolites with a P-value < 0.01 were considered differentially expressed metabolites between the two groups. In total, 194 metabolites were identified in positive ion mode (Supplementary Table 1), and 295 were identified in negative ion mode (Supplementary Table 2).

Further variance analysis was performed on metabolites from the recurrence, preoperative, three-month postoperative, and six-month postoperative groups, which matched those identified in the previous step (Fig. 2c). The F-value was utilized to evaluate inter-group differences, with higher F-values indicating a better equation fitting. A F-value close to 1 indicated no statistical significance, while values greater than 1 indicated statistically significant differences between groups. Metabolites with F-values > 1 and P-values < 0.01 were considered differentially expressed, leading to the identification of 418 metabolites (Supplementary Table 3).

### Differential metabolite analysis and group classification in gastric cancer progression

**Fig. 3:**
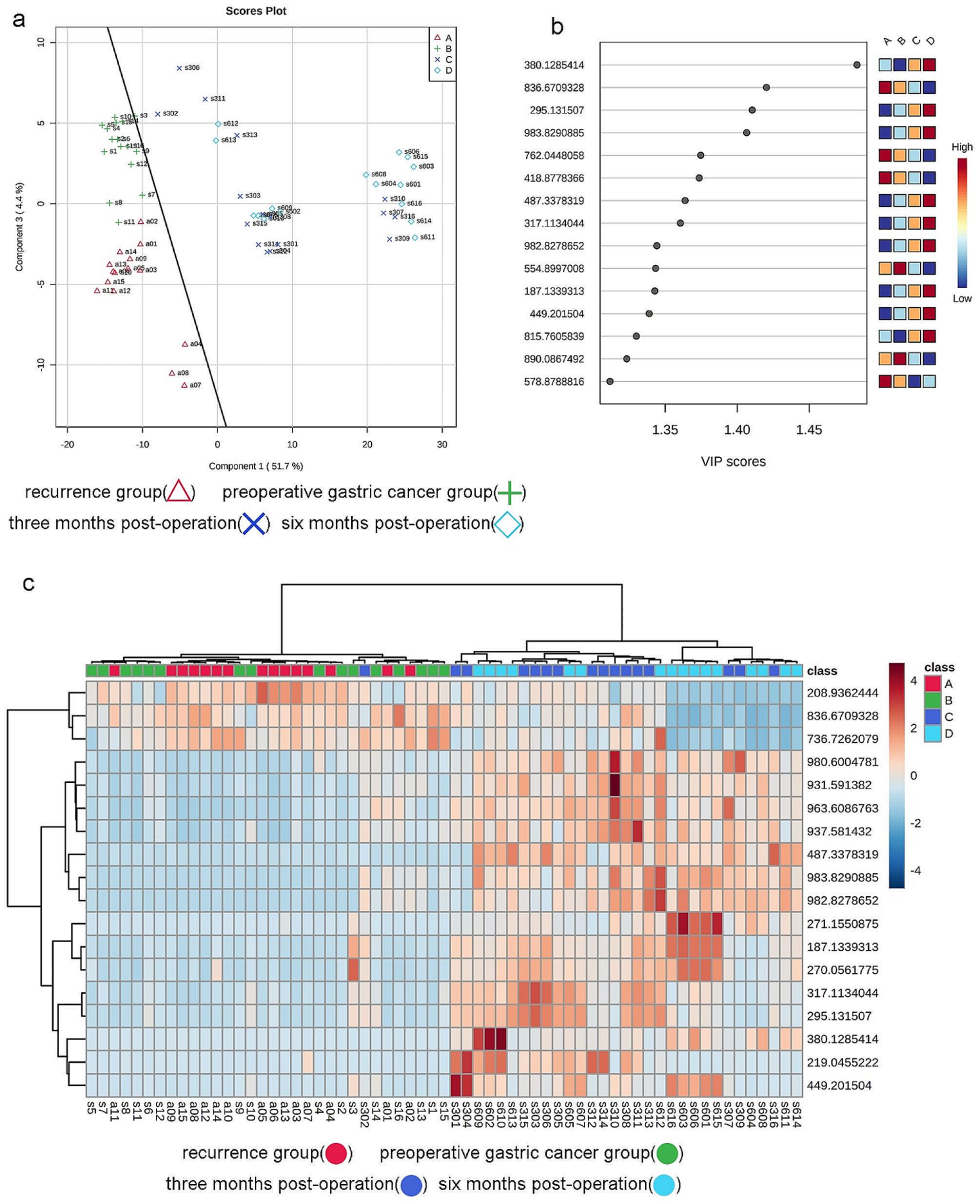
PLS-DA Plot, VIP Score Plot, and Clustering Analysis of 18 Differential Metabolites. (**a**) PLS-DA plot; (**b**) VIP (Variable Importance in Projection) score plot for the selected differential metabolites with VIP > 1.2; (**c**) Hierarchical clustering analysis of the 18 differential metabolites. The color scale represents the relative abundance of metabolites, with red indicating higher abundance, deep blue indicating lower abundance, and light blue indicating zero abundance. The right side of the figure shows the mass-to-charge ratio (m/z) of each metabolite, while the dendrogram on the left and top represents the clustering results of the differential metabolites. The sample numbers are shown at the bottom of the figure

To assess the discriminatory ability of these metabolites among the recurrence, preoperative, three-month postoperative, and six-month postoperative groups, PLS-DA was performed on the differential metabolites selected from the variance analysis (Fig. 3a). The results demonstrated distinct separations among the four sample groups, with good clustering within the same group. Based on the PLS-DA results, 18 metabolites with weight values VIP > 1.2 were identified as differential metabolites (Fig. 3b).

Hierarchical clustering analysis was conducted on the identified differential metabolites (Fig. 3c). The color intensity corresponds to the relative peak areas of the metabolites, with similar colors indicating similar peak areas. The relative peak areas of the differential metabolites served as the clustering criterion. The results revealed the presence of differential metabolites among the recurrence, preoperative, three-month postoperative, and six-month postoperative groups. Additionally, the metabolites of the recurrence and preoperative groups might exhibit similarities, as could the metabolites of the three-month postoperative and six-month postoperative groups. The recurrence and preoperative groups clustered on the left side, while the three-month postoperative and six-month postoperative groups clustered on the right side. Therefore, the four sample groups could be divided into two main groups: the tumor-bearing group (comprising the original recurrence and preoperative groups) and the tumor-free group (comprising the original three-month postoperative and six-month postoperative groups).

### Metabolomic profiling reveals distinctive markers in gastric cancer progression

**Fig. 4:**
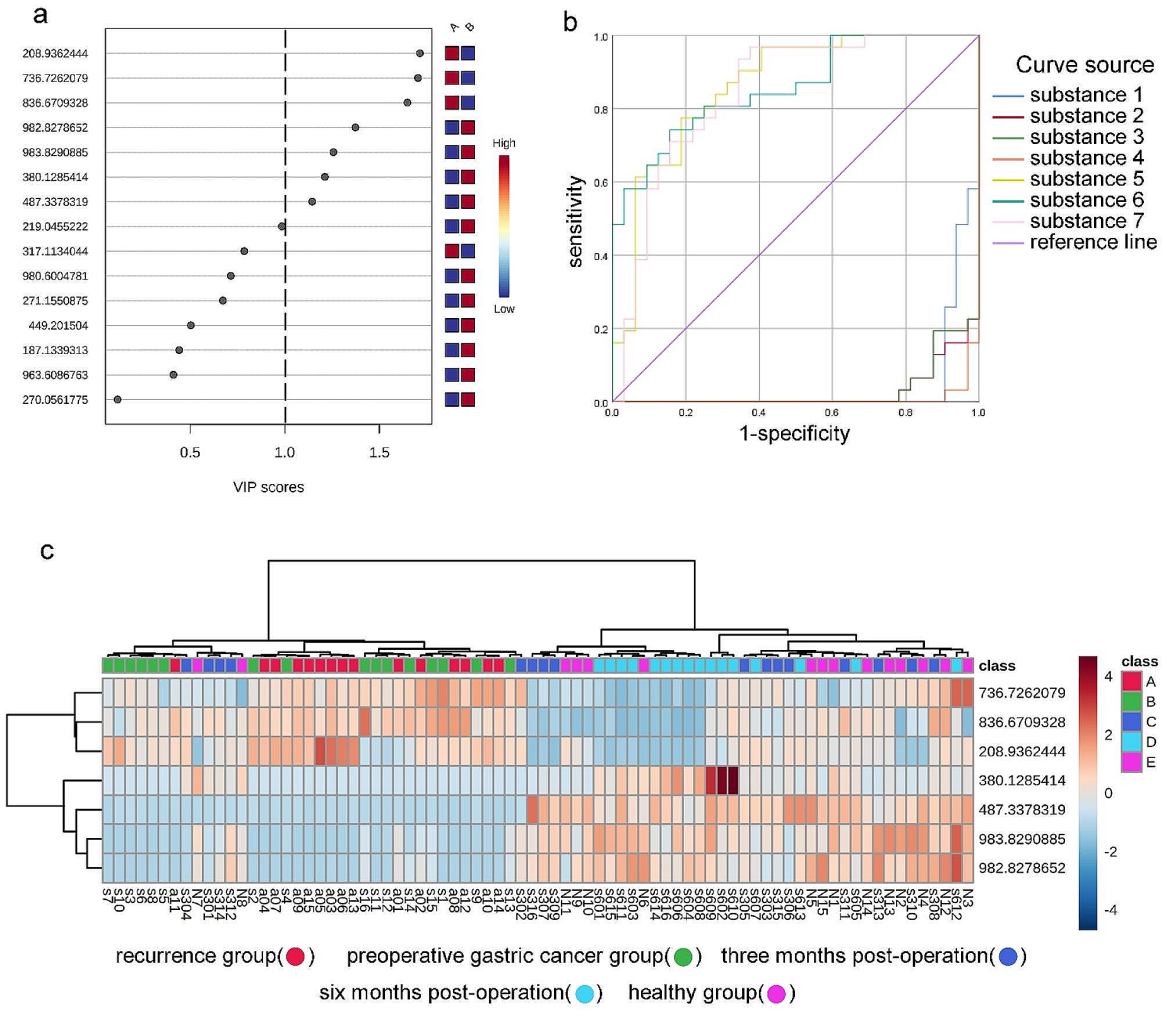
Discriminant Analysis VIP Scores, ROC Curves, and Hierarchical Clustering Analysis of 7 Differential Metabolites among 5 Groups. (**a**) Discriminant analysis VIP score plot of the selected differential metabolites; (**b**) ROC curves. The vertical axis represents sensitivity (true positive rate), and the horizontal axis represents 1-specificity (false positive rate). Each line represents a specific metabolite; (**c**) Hierarchical clustering analysis of the 7 differential metabolites among the Recurrence Group, Preoperative Group, Three-month Postoperative Group, Six-month Postoperative Group, and Healthy Control Group. Red color indicates metabolites with higher peak intensities, deep blue color represents metabolites with lower peak intensities, and light blue color indicates metabolites with zero peak intensities. The color intensity corresponds to the peak intensity of the metabolites. The right side of the figure displays the mass-to-charge ratios of each metabolite, while the left and upper dendrograms represent the clustering results of the differential metabolites. The bottom part of the figure shows the sample numbers

PLS-DA was conducted on the screened differential metabolites, and the VIP score plot was obtained (Fig. 4a). Metabolites with VIP values greater than 1.0 were considered important differential metabolites, and seven metabolites were selected. The mass-to-charge ratio (m/z) of these seven differential metabolites was imported into the HMDB to identify their structures and names (Tables 1 and 2).

**Table 1:** The structures and names of seven identified differential metabolites were determined

Substance Number	Mass-to-charge ratio	Substance	Academic name
Substance 1	487.3378	C25H43O7P	Lysophosphatidic acid
Substance 2	983.8291	C64H120O6	Triglycerides
Substance 3	982.8279	C49H57N9O12	Lysine
Substance 4	380.1285	C18H40NO5P	Sphingosine-1-phosphate
Substance 5	836.6709	C48H88NO8P	Phosphatidylcholine
Substance 6	208.9362	C40H71NO4	Oxidized ceramide
Substance 7	736.7262	C37H71O12P	Phosphatidylglycerol

**Table 2:** The sensitivity and specificity of differential metabolites in sample discrimination

Forecast result	Actual statistical results		In total	Sensitivity	Specificity
	Tumor group	No tumor group			
Tumor group	31	4	35	100%	87.5%
No tumor group	0	28	28		
In total	31	32	63		

ROC curves were generated to assess the accuracy of these differential metabolites (Fig. 4b). The area under the ROC curve (AUC) values (Table 3) were used to evaluate the sensitivity of these differential metabolites in distinguishing between the tumor-bearing and tumor-free groups. A curve closer to the top-left corner indicated a higher abundance of the corresponding metabolite in the tumor-bearing group, while a curve closer to the bottom-right corner indicated a higher abundance in the tumor-free group. Metabolites with AUC values > 0.8 or < 0.1 were considered potentially important differential metabolites. Seven differential metabolites were selected, including Lysophosphatidic acid, triglycerides, lysine, sphingosine-1-phosphate, phosphatidylcholine, oxidized ceramide, and phosphatidylglycerol. These metabolites exhibited significant differences between the tumor-bearing and tumor-free groups, effectively distinguishing between the two groups.

**Table 3:** Area under the ROC curve

Test outcome variable	Area	Standard error	Asymptotically significant	Asymptotic 95% confidence interval Lower limitUpper limit	
				Lower limit	Upper limit
Substance 1	0.041	0.025	0.000	0.000	0.091
Substance 2	0.026	0.015	0.000	0.000	0.056
Substance 3	0.030	0.017	0.000	0.000	0.063
Substance 4	0.007	0.007	0.000	0.000	0.020
Substance 5	0.861	0.047	0.000	0.769	0.953
Substance 6	0.857	0.046	0.000	0.766	0.947
Substance 7	0.844	0.051	0.000	0.744	0.943

The final seven selected differential metabolites were subjected to hierarchical clustering analysis in the recurrence, preoperative, three-month postoperative, six-month postoperative, and healthy control groups (Fig. 4c). The results indicated the presence of distinct differential metabolites among the five sample groups, with some metabolites showing similarities. The recurrence and preoperative groups clustered on the left side of the graph, while the three-month postoperative, six-month postoperative, and healthy control groups mostly clustered on the right side. A small portion of the three-month postoperative and healthy control groups samples were distributed within the recurrence and preoperative groups, possibly due to experimental variations and other objective factors. Overall, the recurrence and preoperative groups were merged, while the three-month postoperative, six-month postoperative, and healthy control groups were also grouped together.

### Mean plots of seven differential metabolites among different groups

**Fig. 5:**
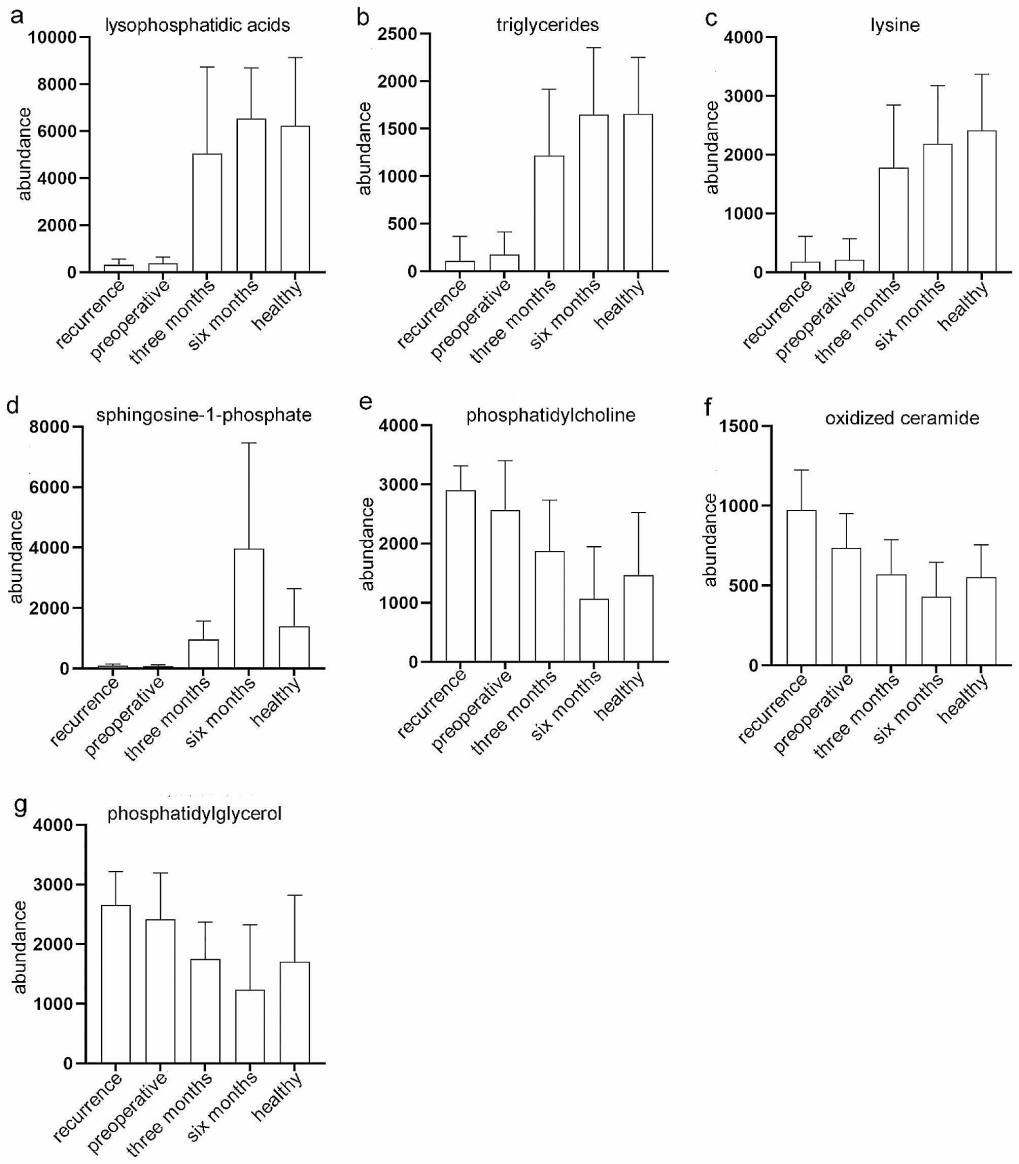
Mean Plots of 7 Differential Metabolites Among Five Groups. The plots depict the mean abundance of 7 differential metabolites among the five groups: Recurrence Group, Preoperative Group, Three-month Postoperative Group, Six-month Postoperative Group, and Healthy Control Group. The y-axis represents the relative abundance of the metabolites, while the x-axis shows the different groups. Panels (**a**) to (**g**) represent the following metabolites: (**a**) Lysophosphatidic acids; (**b**) Triglycerides; (**c**) Lysine; (**d**) Sphingosine-1-phosphate; (**e**) Phosphatidylcholine; (**f**) Oxidized Ceramide and (**g**) Phosphatidylglycerol

We plotted the mean abundance of the selected seven differential metabolites among the Recurrence Group, Preoperative Group, Three-month Postoperative Group, Six-month Postoperative Group, and Healthy Control Group (Fig. 5a-5g) to observe the changes in the abundance of these metabolites across each group. The results showed that the abundance of Lysophosphatidic acids, triglycerides, lysine, and sphingosine-1-phosphate was significantly higher in the “Three-month Postoperative Group,” “Six-month Postoperative Group,” and “Healthy Control Group” compared to the “Preoperative Group” and “Recurrence Group.” On the other hand, phosphatidylcholine, oxidized ceramide, and phosphatidylglycerol had significantly lower abundances in the “Three-month Postoperative Group,” “Six-month Postoperative Group,” and “Healthy Control Group” compared to the “Preoperative Group” and “Recurrence Group.” Additionally, there were no significant differences in the abundance of these metabolites between the “Preoperative Group” and “Recurrence Group,” as well as between the “Three-month Postoperative Group,” “Six-month Postoperative Group,” and “Healthy Control Group.”

## Discussion

Gastric cancer ranks as the fifth most diagnosed malignancy globally, with over one million new cases reported annually [14]. The lack of effective early diagnostic methods often results in late-stage diagnoses, leading to higher mortality rates among gastric cancer patients [14]. While radiotherapy and chemotherapy may improve outcomes, curative treatment largely relies on surgery-based multimodal therapies [15]. Hence, enhancing the effectiveness of gastric cancer surgery and reducing postoperative recurrence have become paramount. Currently, tumor staging and prognosis evaluation methods have limitations [16–18]. However, recent advancements in metabolomics offer new opportunities for identifying novel cancer diagnostic markers and prognostic assessments [19–21].

In this study, we investigated the serum samples collected from 15 healthy individuals, 16 gastric cancer patients before surgery, 16 patients at 3 months after surgery, 16 patients at 6 months after surgery, and 15 patients with gastric cancer recurrence using LC-MS. Our findings revealed significant alterations in the abundance of several metabolites among the different groups. Specifically, lysophosphatidic acids, triglycerides, lysine, and sphingosine-1-phosphate showed elevated levels in the three-month postoperative, six-month postoperative, and healthy control groups compared to the preoperative and recurrence groups. Conversely, phosphatidylcholine, oxidized ceramide, and phosphatidylglycerol exhibited decreased levels in the three-month postoperative, six-month postoperative, and healthy control groups compared to the preoperative and recurrence groups. Notably, no significant differences were observed between the preoperative and recurrence groups, nor between the three-month postoperative, six-month postoperative, and healthy control groups.

Among the identified metabolites, triglycerides are recognized as essential blood lipids involved in energy storage, signaling pathways, and structural composition. Lipid metabolism dysregulation represents one of the most prominent metabolic alterations in cancer. Cancer cells exploit lipid metabolism to facilitate proliferation, survival, invasion, and metastasis and to influence the tumor microenvironment, providing energy, membrane components, and signaling molecules required for cancer progression and treatment [22]. Prior studies have shown that the preoperative ratio of triglycerides to high-density lipoprotein cholesterol is an effective and independent prognostic factor for predicting 5-year mortality and improving prognosis in gastric cancer patients [22, 23].

Phosphatidic acid, phosphatidylcholine, and phosphatidylglycerol are all glycerophospholipids, critical constituents of the cellular lipid bilayer involved in metabolism and signal transduction. Phosphatidic acid signaling is vital in tumor-induced inflammation, promoting cancer progression, metastasis, and fibrosis [24]. Additionally, inflammation and increased phosphatidic acid signaling contribute to immune evasion, thereby protecting cancer cells from immune system destruction [24].

Lysine is associated with post-translational modifications, particularly lysine methylation, a common post-translational modification. Notably, lactate dehydrogenase A succinylation at K222 has been shown to inhibit its degradation, promoting invasion and proliferation in gastric cancer [25]. Lysine may exert its oncogenic effects through other histone or non-histone substrates, or non-enzymatic protein lysine methyltransferase activation may stimulate carcinogenic mechanisms in specific cancer types [26]. Sphingolipids comprise a series of novel lipid bio-regulators that play crucial roles in maintaining barrier function and fluidity and regulating multiple signals during tumorigenesis [27]. Both sphingosine-1-phosphate and Lysophosphatidic acids have been implicated in tumor immune responses [28].

Oxidized ceramides represent the oxidative form of ceramides, which are vital precursors of sphingolipids involved in cell cycle, differentiation, aging, and apoptosis processes. Oxidized ceramides play significant roles in cancer development and treatment [29]. They have emerged as potent tumor suppressors and garnered attention for their potential application in combined therapies for cancer treatment [24]. Various factors, such as chemotherapy drugs, cytotoxic agents, hypoxic microenvironments, malnutrition, radiation, and hyperthermia, promote apoptosis in tumor cells by increasing enzymatic activity related to the synthesis of ceramides [30, 31]. Recent studies have underscored the significant impact of interfering with ceramide production and metabolism on cancer treatment, highlighting their potential as signaling molecules with antiproliferative and pro-apoptotic effects [32, 33].

The observed alterations in the abundance of these metabolites in the serum of gastric cancer patients suggest their potential as diagnostic and prognostic biomarkers, warranting further investigation to validate their clinical relevance. The present study provides valuable insights into the metabolic changes associated with gastric cancer and postoperative monitoring, offering new avenues for improved clinical management and patient outcomes. These findings may pave the way for developing targeted therapeutic strategies and personalized treatment approaches, ultimately benefiting gastric cancer patients by facilitating early detection and more effective recurrence monitoring. Continued research in this area has the potential to revolutionize the field of gastric cancer management and contribute to the advancement of precision medicine.

## Conclusion

This study focused on identifying differential metabolites in serum samples between healthy individuals and gastric cancer patients. Among the metabolites analyzed, we have identified seven potential biomarkers, namely, hemolytic phospholipid acid, triglycerides, lysine, Sphingosine-1-phosphate, phosphatidylcholine, oxidized ceramide, and phosphatidylglycerol. These differential metabolites are promising biomarkers for evaluating the efficacy of postoperative treatments and monitoring cancer recurrence in gastric cancer patients. However, further research is warranted to validate their roles in gastric cancer by conducting functional and clinical sample analyses of the metabolic pathways involved. Comprehensive validation studies will provide more robust evidence for the clinical utility of these biomarkers, bringing us closer to their translation into clinical practice for improved patient care and outcomes.

### Electronic supplementary material

Below is the link to the electronic supplementary material.

Supplementary Material 1: 194 metabolites identified in positive ion mode

Supplementary Material 2: 295 metabolites identified in negative ion mode

Supplementary Material 3:The identification of 418 metabolites

## Data Availability

The authors confirm that the data supporting the findings of this study are available within the article. Raw data that support the findings of the study are available from the corresponding author, upon reasonable request.
