# Supplementary material for: Utilizing serum metabolomics for assessing postoperative efficacy and monitoring recurrence in gastric cancer patients
Source: BMC Cancer. 2024 Jan 2;24:27. doi: 10.1186/s12885-023-11786-2 (PMC10763142; doi:10.1186/s12885-023-11786-2)
Supplement: Supplementary file 1 — Supplementary Material 1: 194 metabolites identified in positive ion mode [file 12885_2023_11786_MOESM1_ESM.docx]

Supplementary table1. 194 metabolites identified in positive ion mode

| Ion Mode | Mass-to-charge ratio | P |
| --- | --- | --- |
| ESI+ | 738.4239211 | 4.01E-11 |
| ESI+ | 740.4297011 | 4.25E-11 |
| ESI+ | 870.5237867 | 1.43E-10 |
| ESI+ | 282.2783452 | 2.58E-10 |
| ESI+ | 443.2710671 | 2.66E-10 |
| ESI+ | 282.2786713 | 4.16E-10 |
| ESI+ | 663.3351846 | 1.12E-09 |
| ESI+ | 757.3968312 | 1.89E-09 |
| ESI+ | 479.2843297 | 3.02E-09 |
| ESI+ | 880.9895855 | 3.21E-09 |
| ESI+ | 487.3378319 | 3.50E-09 |
| ESI+ | 652.2204891 | 4.65E-09 |
| ESI+ | 668.315585 | 5.82E-09 |
| ESI+ | 930.547663 | 6.37E-09 |
| ESI+ | 1106.651899 | 1.05E-08 |
| ESI+ | 631.7427877 | 3.02E-08 |
| ESI+ | 145.0163565 | 3.28E-08 |
| ESI+ | 853.517025 | 3.92E-08 |
| ESI+ | 403.0913559 | 4.46E-08 |
| ESI+ | 770.7262352 | 5.33E-08 |
| ESI+ | 854.4690499 | 5.77E-08 |
| ESI+ | 682.2919406 | 6.82E-08 |
| ESI+ | 848.5114666 | 8.32E-08 |
| ESI+ | 1239.734718 | 8.41E-08 |
| ESI+ | 1194.702677 | 1.21E-07 |
| ESI+ | 517.3336732 | 1.22E-07 |
| ESI+ | 587.0079076 | 1.25E-07 |
| ESI+ | 516.3301468 | 3.89E-07 |
| ESI+ | 444.1850279 | 4.40E-07 |
| ESI+ | 565.0244005 | 5.23E-07 |
| ESI+ | 861.4543389 | 5.85E-07 |
| ESI+ | 598.3575933 | 7.10E-07 |
| ESI+ | 405.3673136 | 8.70E-07 |
| ESI+ | 576.8819352 | 9.50E-07 |
| ESI+ | 626.9766675 | 1.01E-06 |
| ESI+ | 725.0629315 | 1.10E-06 |
| ESI+ | 460.9651971 | 1.30E-06 |
| ESI+ | 853.9680475 | 1.39E-06 |
| ESI+ | 585.3576577 | 1.71E-06 |
| ESI+ | 738.9481312 | 2.00E-06 |
| ESI+ | 290.2685559 | 2.11E-06 |
| ESI+ | 759.3628711 | 2.34E-06 |
| ESI+ | 858.9768341 | 2.43E-06 |
| ESI+ | 722.059916 | 2.46E-06 |
| ESI+ | 1198.905299 | 2.52E-06 |
| ESI+ | 682.3610126 | 2.52E-06 |
| ESI+ | 761.0410084 | 2.59E-06 |
| ESI+ | 1066.629203 | 2.67E-06 |
| ESI+ | 967.5819532 | 2.96E-06 |
| ESI+ | 716.4337901 | 3.07E-06 |
| ESI+ | 516.3303522 | 3.22E-06 |
| ESI+ | 808.4671154 | 3.49E-06 |
| ESI+ | 651.3976837 | 4.02E-06 |
| ESI+ | 449.3440607 | 4.84E-06 |
| ESI+ | 446.2520453 | 4.94E-06 |
| ESI+ | 739.0602596 | 5.39E-06 |
| ESI+ | 710.3929973 | 6.02E-06 |
| ESI+ | 417.0010872 | 6.54E-06 |
| ESI+ | 611.005061 | 6.56E-06 |
| ESI+ | 443.1814412 | 6.65E-06 |
| ESI+ | 509.3101327 | 6.89E-06 |
| ESI+ | 694.9223372 | 7.00E-06 |
| ESI+ | 871.1110327 | 7.91E-06 |
| ESI+ | 646.7102642 | 8.05E-06 |
| ESI+ | 586.3614314 | 8.45E-06 |
| ESI+ | 1091.302646 | 8.79E-06 |
| ESI+ | 129.0383968 | 9.16E-06 |
| ESI+ | 805.3457704 | 9.23E-06 |
| ESI+ | 754.2563787 | 9.48E-06 |
| ESI+ | 1166.700845 | 9.69E-06 |
| ESI+ | 807.9651686 | 9.76E-06 |
| ESI+ | 716.9349885 | 1.04E-05 |
| ESI+ | 156.0488752 | 1.08E-05 |
| ESI+ | 811.2761162 | 1.11E-05 |
| ESI+ | 457.405138 | 1.13E-05 |
| ESI+ | 654.3376976 | 1.16E-05 |
| ESI+ | 755.0337351 | 1.39E-05 |
| ESI+ | 219.1702841 | 1.41E-05 |
| ESI+ | 780.5505226 | 1.64E-05 |
| ESI+ | 146.1804919 | 1.75E-05 |
| ESI+ | 859.0195608 | 1.87E-05 |
| ESI+ | 610.8259064 | 1.90E-05 |
| ESI+ | 460.2082752 | 1.91E-05 |
| ESI+ | 1091.178316 | 2.25E-05 |
| ESI+ | 546.0468957 | 2.40E-05 |
| ESI+ | 890.0867492 | 3.12E-05 |
| ESI+ | 959.0776713 | 3.61E-05 |
| ESI+ | 1062.153443 | 4.61E-05 |
| ESI+ | 411.1665539 | 6.77E-05 |
| ESI+ | 662.4666206 | 7.51E-05 |
| ESI+ | 513.3183345 | 7.76E-05 |
| ESI+ | 842.7733534 | 8.34E-05 |
| ESI+ | 793.9401713 | 8.44E-05 |
| ESI+ | 128.0688513 | 8.65E-05 |
| ESI+ | 810.4445313 | 8.97E-05 |
| ESI+ | 498.9383469 | 9.02E-05 |
| ESI+ | 545.3393141 | 9.31E-05 |
| ESI+ | 707.4952391 | 9.67E-05 |
| ESI+ | 620.8741449 | 9.70E-05 |
| ESI+ | 881.9900999 | 9.72E-05 |
| ESI+ | 655.7808499 | 0.00010031 |
| ESI+ | 762.0448058 | 0.00010282 |
| ESI+ | 512.9147128 | 0.00010441 |
| ESI+ | 571.0320128 | 0.00010761 |
| ESI+ | 531.3224227 | 0.00010861 |
| ESI+ | 554.8997008 | 0.00010945 |
| ESI+ | 291.2718193 | 0.00011032 |
| ESI+ | 777.0161563 | 0.00011968 |
| ESI+ | 815.6937326 | 0.00012425 |
| ESI+ | 368.6252644 | 0.00012456 |
| ESI+ | 660.8576919 | 0.00012485 |
| ESI+ | 332.2700757 | 0.00012605 |
| ESI+ | 618.1787267 | 0.00012891 |
| ESI+ | 695.9249826 | 0.00013299 |
| ESI+ | 916.2285128 | 0.00013373 |
| ESI+ | 578.8788816 | 0.00013634 |
| ESI+ | 510.3177673 | 0.00014258 |
| ESI+ | 149.0225273 | 0.00014385 |
| ESI+ | 733.051547 | 0.00014538 |
| ESI+ | 729.3753232 | 0.00014604 |
| ESI+ | 470.4562872 | 0.00014814 |
| ESI+ | 116.8894554 | 0.00015727 |
| ESI+ | 669.3071578 | 0.00015923 |
| ESI+ | 350.2891017 | 0.0001593 |
| ESI+ | 558.9962243 | 0.00016064 |
| ESI+ | 399.3242098 | 0.00016142 |
| ESI+ | 414.3565223 | 0.00016379 |
| ESI+ | 699.4075865 | 0.00019698 |
| ESI+ | 402.2368654 | 0.00019723 |
| ESI+ | 703.0825565 | 0.00020139 |
| ESI+ | 469.2915145 | 0.00020281 |
| ESI+ | 884.6062507 | 0.00020849 |
| ESI+ | 801.3866271 | 0.00021775 |
| ESI+ | 1087.558185 | 0.0002248 |
| ESI+ | 710.2740142 | 0.00022586 |
| ESI+ | 284.1844533 | 0.00024668 |
| ESI+ | 398.2552178 | 0.00024896 |
| ESI+ | 824.41253 | 0.00025329 |
| ESI+ | 372.1760189 | 0.00025859 |
| ESI+ | 760.9615185 | 0.00025892 |
| ESI+ | 831.9638324 | 0.00025914 |
| ESI+ | 1075.112668 | 0.00027545 |
| ESI+ | 361.0475452 | 0.00028299 |
| ESI+ | 780.8054129 | 0.00029741 |
| ESI+ | 783.0242575 | 0.00030085 |
| ESI+ | 441.4223124 | 0.00030192 |
| ESI+ | 696.3754671 | 0.00030371 |
| ESI+ | 793.4378169 | 0.00030434 |
| ESI+ | 293.0983193 | 0.00031678 |
| ESI+ | 1330.933426 | 0.00032122 |
| ESI+ | 553.2535947 | 0.00032219 |
| ESI+ | 462.9620789 | 0.00033484 |
| ESI+ | 415.2106128 | 0.00035265 |
| ESI+ | 1053.132655 | 0.00035269 |
| ESI+ | 418.8778366 | 0.00035302 |
| ESI+ | 477.1021659 | 0.00035911 |
| ESI+ | 369.0082893 | 0.00036455 |
| ESI+ | 827.0010669 | 0.00036888 |
| ESI+ | 511.3213837 | 0.0003986 |
| ESI+ | 522.3556167 | 0.00040083 |
| ESI+ | 376.6236402 | 0.00040619 |
| ESI+ | 447.2907482 | 0.00041513 |
| ESI+ | 681.0843981 | 0.00043624 |
| ESI+ | 648.4511723 | 0.00044615 |
| ESI+ | 545.3419681 | 0.00045443 |
| ESI+ | 485.5024417 | 0.00045727 |
| ESI+ | 453.1660051 | 0.000464 |
| ESI+ | 597.8161869 | 0.00046764 |
| ESI+ | 206.099734 | 0.00047519 |
| ESI+ | 189.0959334 | 0.00048501 |
| ESI+ | 708.644113 | 0.00049543 |
| ESI+ | 504.3345878 | 0.00051301 |
| ESI+ | 953.1748561 | 0.00055189 |
| ESI+ | 860.1172304 | 0.00056251 |
| ESI+ | 578.8684713 | 0.00056567 |
| ESI+ | 319.2616298 | 0.00061087 |
| ESI+ | 112.8946828 | 0.00061217 |
| ESI+ | 438.9831485 | 0.00062744 |
| ESI+ | 217.951195 | 0.00063079 |
| ESI+ | 680.7510502 | 0.00065626 |
| ESI+ | 144.0798704 | 0.00069054 |
| ESI+ | 740.0632553 | 0.00069924 |
| ESI+ | 629.8848541 | 0.00071003 |
| ESI+ | 630.1539011 | 0.00071678 |
| ESI+ | 124.0859101 | 0.00071996 |
| ESI+ | 527.3204947 | 0.00074079 |
| ESI+ | 147.0624301 | 0.00074254 |
| ESI+ | 629.1511758 | 0.00074283 |
| ESI+ | 638.8131206 | 0.00075405 |
| ESI+ | 928.7476463 | 0.00077204 |
| ESI+ | 527.3202236 | 0.00078624 |
| ESI+ | 401.3444563 | 0.00079112 |
| ESI+ | 578.6957356 | 0.00080546 |
| ESI+ | 792.9382063 | 0.0008205 |
