# Supplementary material for: Utilizing serum metabolomics for assessing postoperative efficacy and monitoring recurrence in gastric cancer patients
Source: BMC Cancer. 2024 Jan 2;24:27. doi: 10.1186/s12885-023-11786-2 (PMC10763142; doi:10.1186/s12885-023-11786-2)
Supplement: Supplementary file 2 — Supplementary Material 2: 295 metabolites identified in negative ion mode [file 12885_2023_11786_MOESM2_ESM.docx]

Supplementary table2. 295 metabolites identified in negative ion mode

| Ion Mode | Mass-to-charge ratio | P |
| --- | --- | --- |
| ESI- | 675.2470844 | 1.14E-11 |
| ESI- | 656.3171663 | 2.29E-10 |
| ESI- | 517.0975415 | 1.12E-09 |
| ESI- | 846.8098981 | 3.01E-09 |
| ESI- | 548.1950099 | 5.68E-09 |
| ESI- | 461.2019173 | 8.51E-09 |
| ESI- | 815.7605839 | 8.84E-09 |
| ESI- | 1192.635818 | 1.13E-08 |
| ESI- | 377.2285342 | 1.33E-08 |
| ESI- | 380.1285414 | 1.40E-08 |
| ESI- | 709.3418165 | 1.66E-08 |
| ESI- | 369.9072839 | 1.99E-08 |
| ESI- | 371.9045924 | 2.97E-08 |
| ESI- | 605.3199146 | 3.05E-08 |
| ESI- | 983.8290885 | 5.60E-08 |
| ESI- | 271.1550875 | 6.29E-08 |
| ESI- | 164.8360293 | 9.71E-08 |
| ESI- | 982.8278652 | 1.15E-07 |
| ESI- | 473.0708416 | 2.19E-07 |
| ESI- | 944.4988877 | 2.54E-07 |
| ESI- | 463.2177709 | 2.83E-07 |
| ESI- | 1193.639312 | 3.21E-07 |
| ESI- | 917.8393336 | 3.61E-07 |
| ESI- | 665.2593908 | 5.34E-07 |
| ESI- | 514.1305494 | 5.49E-07 |
| ESI- | 204.086072 | 6.29E-07 |
| ESI- | 1149.654532 | 6.64E-07 |
| ESI- | 1474.761351 | 6.70E-07 |
| ESI- | 293.1757228 | 8.69E-07 |
| ESI- | 819.7876443 | 1.01E-06 |
| ESI- | 1125.654096 | 1.21E-06 |
| ESI- | 1124.65261 | 1.26E-06 |
| ESI- | 429.1532417 | 1.27E-06 |
| ESI- | 716.8520311 | 1.31E-06 |
| ESI- | 219.0455222 | 1.56E-06 |
| ESI- | 243.114125 | 2.05E-06 |
| ESI- | 245.139468 | 2.07E-06 |
| ESI- | 886.3826687 | 2.12E-06 |
| ESI- | 425.9411928 | 2.22E-06 |
| ESI- | 524.0390736 | 3.06E-06 |
| ESI- | 1090.920515 | 3.22E-06 |
| ESI- | 187.1339313 | 4.22E-06 |
| ESI- | 284.7746255 | 4.64E-06 |
| ESI- | 945.0003138 | 5.25E-06 |
| ESI- | 270.0561775 | 5.65E-06 |
| ESI- | 376.2252092 | 5.88E-06 |
| ESI- | 1308.189999 | 5.89E-06 |
| ESI- | 444.2120691 | 6.25E-06 |
| ESI- | 350.2097076 | 6.86E-06 |
| ESI- | 673.3075029 | 7.02E-06 |
| ESI- | 451.1083783 | 7.34E-06 |
| ESI- | 945.5026095 | 7.38E-06 |
| ESI- | 145.0616326 | 7.99E-06 |
| ESI- | 943.9972726 | 8.12E-06 |
| ESI- | 522.0414998 | 8.31E-06 |
| ESI- | 1427.752287 | 1.05E-05 |
| ESI- | 438.2111411 | 1.18E-05 |
| ESI- | 1091.921926 | 1.23E-05 |
| ESI- | 981.2382584 | 1.37E-05 |
| ESI- | 441.210079 | 1.42E-05 |
| ESI- | 862.3952328 | 1.57E-05 |
| ESI- | 964.32641 | 1.58E-05 |
| ESI- | 445.1267624 | 1.63E-05 |
| ESI- | 439.2198887 | 1.79E-05 |
| ESI- | 141.1281715 | 1.88E-05 |
| ESI- | 661.2523035 | 2.81E-05 |
| ESI- | 950.1560143 | 2.86E-05 |
| ESI- | 651.3583457 | 2.92E-05 |
| ESI- | 264.1070964 | 3.07E-05 |
| ESI- | 116.050258 | 3.14E-05 |
| ESI- | 672.2431498 | 3.39E-05 |
| ESI- | 697.2707822 | 3.50E-05 |
| ESI- | 812.3926156 | 3.55E-05 |
| ESI- | 492.1542762 | 3.70E-05 |
| ESI- | 1480.269643 | 4.04E-05 |
| ESI- | 491.1231739 | 4.23E-05 |
| ESI- | 457.2427345 | 4.41E-05 |
| ESI- | 1450.755485 | 4.47E-05 |
| ESI- | 427.9385207 | 4.55E-05 |
| ESI- | 1108.594639 | 4.83E-05 |
| ESI- | 855.6589832 | 4.92E-05 |
| ESI- | 321.061912 | 4.95E-05 |
| ESI- | 423.9443855 | 5.27E-05 |
| ESI- | 1107.592776 | 5.39E-05 |
| ESI- | 580.5576246 | 5.43E-05 |
| ESI- | 1391.205541 | 5.57E-05 |
| ESI- | 1451.256989 | 5.71E-05 |
| ESI- | 418.2147242 | 6.30E-05 |
| ESI- | 1451.757917 | 6.39E-05 |
| ESI- | 963.6086763 | 6.83E-05 |
| ESI- | 994.1188454 | 6.98E-05 |
| ESI- | 405.91547 | 7.16E-05 |
| ESI- | 717.3532354 | 7.47E-05 |
| ESI- | 438.0776193 | 7.54E-05 |
| ESI- | 292.0868397 | 8.44E-05 |
| ESI- | 696.2626722 | 8.48E-05 |
| ESI- | 1086.68092 | 9.08E-05 |
| ESI- | 722.7506948 | 9.99E-05 |
| ESI- | 255.1215167 | 0.0001009 |
| ESI- | 618.2378507 | 0.00010588 |
| ESI- | 408.9322768 | 0.00010919 |
| ESI- | 918.6073853 | 0.00011092 |
| ESI- | 111.0085109 | 0.00011098 |
| ESI- | 460.0603056 | 0.00011572 |
| ESI- | 188.1372673 | 0.00011905 |
| ESI- | 272.111143 | 0.00012047 |
| ESI- | 317.1134044 | 0.00012192 |
| ESI- | 1473.759096 | 0.00012919 |
| ESI- | 368.9712519 | 0.00013135 |
| ESI- | 474.8668976 | 0.00013166 |
| ESI- | 658.0662241 | 0.00013368 |
| ESI- | 477.1253728 | 0.00013703 |
| ESI- | 645.2686642 | 0.00014181 |
| ESI- | 1071.443807 | 0.00014603 |
| ESI- | 729.2684427 | 0.00015304 |
| ESI- | 508.1136447 | 0.00015564 |
| ESI- | 724.3836627 | 0.00015714 |
| ESI- | 540.8202198 | 0.00015902 |
| ESI- | 666.0947721 | 0.00015951 |
| ESI- | 524.2264462 | 0.0001638 |
| ESI- | 673.245612 | 0.00016873 |
| ESI- | 510.2292082 | 0.00017011 |
| ESI- | 651.1226646 | 0.00017033 |
| ESI- | 966.3313535 | 0.00017149 |
| ESI- | 993.7750452 | 0.0001737 |
| ESI- | 942.2121383 | 0.00017498 |
| ESI- | 605.1547569 | 0.00018174 |
| ESI- | 954.7653202 | 0.00018379 |
| ESI- | 644.8490276 | 0.00018627 |
| ESI- | 419.8957956 | 0.00018833 |
| ESI- | 774.2233471 | 0.00019619 |
| ESI- | 534.2665193 | 0.00019634 |
| ESI- | 1061.16267 | 0.00019996 |
| ESI- | 649.8535626 | 0.00020255 |
| ESI- | 127.0510782 | 0.00021318 |
| ESI- | 435.8804597 | 0.00021416 |
| ESI- | 618.2375536 | 0.00023702 |
| ESI- | 446.1489498 | 0.00023955 |
| ESI- | 430.1563819 | 0.00024027 |
| ESI- | 664.7579012 | 0.00024319 |
| ESI- | 931.591382 | 0.00024367 |
| ESI- | 738.7250624 | 0.00024388 |
| ESI- | 363.0275362 | 0.00025269 |
| ESI- | 527.2138706 | 0.00025534 |
| ESI- | 630.230025 | 0.00025699 |
| ESI- | 893.1385977 | 0.00025705 |
| ESI- | 1429.255391 | 0.00026798 |
| ESI- | 845.0604786 | 0.0002681 |
| ESI- | 1428.747583 | 0.00026842 |
| ESI- | 1277.262386 | 0.00028012 |
| ESI- | 251.0925178 | 0.00028363 |
| ESI- | 1468.812201 | 0.00028736 |
| ESI- | 307.9237434 | 0.00028792 |
| ESI- | 695.7951419 | 0.00029249 |
| ESI- | 393.060644 | 0.00029865 |
| ESI- | 417.2118315 | 0.00030193 |
| ESI- | 991.3421449 | 0.00030453 |
| ESI- | 661.7538089 | 0.00030492 |
| ESI- | 627.5549541 | 0.00030691 |
| ESI- | 963.7552667 | 0.00031157 |
| ESI- | 1088.257302 | 0.00031366 |
| ESI- | 693.2488911 | 0.00031435 |
| ESI- | 552.9803889 | 0.00031891 |
| ESI- | 1452.261464 | 0.00033127 |
| ESI- | 392.8910273 | 0.00033309 |
| ESI- | 1395.548125 | 0.00033713 |
| ESI- | 836.6709328 | 0.00034551 |
| ESI- | 628.7272546 | 0.00034978 |
| ESI- | 1007.779943 | 0.00035909 |
| ESI- | 723.2263068 | 0.00036372 |
| ESI- | 294.8030875 | 0.00036868 |
| ESI- | 350.1502391 | 0.00037481 |
| ESI- | 1099.116574 | 0.00037553 |
| ESI- | 399.0619116 | 0.00037586 |
| ESI- | 408.8641953 | 0.00037757 |
| ESI- | 648.8180211 | 0.00037935 |
| ESI- | 704.239406 | 0.00037952 |
| ESI- | 602.5661659 | 0.00039188 |
| ESI- | 1033.1717 | 0.00039954 |
| ESI- | 1237.483264 | 0.00040701 |
| ESI- | 486.0654031 | 0.00041092 |
| ESI- | 942.3444227 | 0.00041208 |
| ESI- | 740.2895834 | 0.0004223 |
| ESI- | 532.2663141 | 0.00042479 |
| ESI- | 482.0935543 | 0.0004287 |
| ESI- | 173.994541 | 0.00044061 |
| ESI- | 128.0351964 | 0.00044937 |
| ESI- | 699.2686542 | 0.0004569 |
| ESI- | 481.0920427 | 0.00046012 |
| ESI- | 1029.774224 | 0.00046297 |
| ESI- | 1045.108681 | 0.00046509 |
| ESI- | 502.8580098 | 0.00046653 |
| ESI- | 937.581432 | 0.000473 |
| ESI- | 1271.746101 | 0.00047478 |
| ESI- | 817.1455331 | 0.00047898 |
| ESI- | 719.2508985 | 0.00048111 |
| ESI- | 1003.568565 | 0.00048946 |
| ESI- | 263.1039434 | 0.00050091 |
| ESI- | 572.8166562 | 0.00050097 |
| ESI- | 1083.144134 | 0.00050197 |
| ESI- | 538.6569165 | 0.00050678 |
| ESI- | 865.4016689 | 0.00051829 |
| ESI- | 697.2704273 | 0.00051885 |
| ESI- | 1474.258482 | 0.00052424 |
| ESI- | 780.7094594 | 0.00052635 |
| ESI- | 634.8889165 | 0.00052851 |
| ESI- | 407.1715387 | 0.00053311 |
| ESI- | 1264.224887 | 0.00053507 |
| ESI- | 341.9992991 | 0.00054757 |
| ESI- | 474.9420313 | 0.0005508 |
| ESI- | 686.2531878 | 0.00055433 |
| ESI- | 273.9597533 | 0.00055645 |
| ESI- | 629.2280406 | 0.00055843 |
| ESI- | 295.131507 | 0.00057091 |
| ESI- | 1072.446125 | 0.00057558 |
| ESI- | 447.1333473 | 0.00058164 |
| ESI- | 680.2279195 | 0.00059269 |
| ESI- | 655.2236088 | 0.00059716 |
| ESI- | 917.6047802 | 0.00061466 |
| ESI- | 1233.298666 | 0.00062793 |
| ESI- | 598.8527959 | 0.00062908 |
| ESI- | 1153.703822 | 0.00063432 |
| ESI- | 1354.171998 | 0.00063855 |
| ESI- | 395.0016668 | 0.00067845 |
| ESI- | 293.1757135 | 0.00070575 |
| ESI- | 131.9154076 | 0.00070958 |
| ESI- | 448.1086591 | 0.00071069 |
| ESI- | 722.2695923 | 0.0007129 |
| ESI- | 944.2147552 | 0.00073308 |
| ESI- | 1028.769098 | 0.00074406 |
| ESI- | 980.6004781 | 0.00074418 |
| ESI- | 208.9362444 | 0.0007519 |
| ESI- | 428.9711536 | 0.00076375 |
| ESI- | 1274.42997 | 0.0007916 |
| ESI- | 767.5733962 | 0.00079185 |
| ESI- | 641.2154505 | 0.00079797 |
| ESI- | 1317.217314 | 0.00081641 |
| ESI- | 962.6065527 | 0.00082293 |
| ESI- | 744.5618597 | 0.00082452 |
| ESI- | 510.7309705 | 0.00087986 |
| ESI- | 649.8026892 | 0.00088307 |
| ESI- | 704.1318698 | 0.0008977 |
| ESI- | 449.201504 | 0.0009081 |
| ESI- | 423.1511886 | 0.00093019 |
| ESI- | 741.0826951 | 0.00095178 |
| ESI- | 193.0871238 | 0.00097246 |
| ESI- | 892.7909195 | 0.00098758 |
| ESI- | 321.9038247 | 0.00099978 |
| ESI- | 471.9807647 | 0.0010138 |
| ESI- | 851.1614109 | 0.0010767 |
| ESI- | 415.1477647 | 0.0010828 |
| ESI- | 822.694587 | 0.001085 |
| ESI- | 716.7669911 | 0.0011035 |
| ESI- | 1315.216418 | 0.0011165 |
| ESI- | 704.767281 | 0.0011241 |
| ESI- | 1134.669524 | 0.0011304 |
| ESI- | 485.2813382 | 0.0011501 |
| ESI- | 532.9009768 | 0.0011511 |
| ESI- | 409.2216835 | 0.0011552 |
| ESI- | 221.0818921 | 0.0011605 |
| ESI- | 971.3637948 | 0.0011999 |
| ESI- | 926.8511654 | 0.0012162 |
| ESI- | 389.049857 | 0.001236 |
| ESI- | 432.186184 | 0.0012498 |
| ESI- | 675.044284 | 0.0012521 |
| ESI- | 220.1468307 | 0.0012573 |
| ESI- | 592.8351266 | 0.0012908 |
| ESI- | 552.7371836 | 0.0013109 |
| ESI- | 638.780563 | 0.0013265 |
| ESI- | 1155.848228 | 0.001333 |
| ESI- | 180.9733947 | 0.0013446 |
| ESI- | 618.5485171 | 0.0013686 |
| ESI- | 372.9011105 | 0.0013744 |
| ESI- | 478.1150746 | 0.0013813 |
| ESI- | 194.0903245 | 0.0013947 |
| ESI- | 736.7262079 | 0.0014053 |
| ESI- | 264.1070778 | 0.0014137 |
| ESI- | 1233.780863 | 0.0015225 |
| ESI- | 638.0934766 | 0.001565 |
| ESI- | 778.7126253 | 0.0015915 |
| ESI- | 1020.500494 | 0.0016277 |
| ESI- | 970.0089299 | 0.0016368 |
| ESI- | 547.3203928 | 0.001646 |
| ESI- | 728.8108871 | 0.0016711 |
| ESI- | 1457.14531 | 0.0016862 |
| ESI- | 268.8010926 | 0.0016925 |
| ESI- | 147.0448532 | 0.0017078 |
| ESI- | 688.783953 | 0.0017085 |
| ESI- | 1432.501701 | 0.0017595 |
| ESI- | 741.2931364 | 0.0017753 |
| ESI- | 791.760528 | 0.0017868 |
| ESI- | 467.2956674 | 0.001803 |
| ESI- | 555.8694294 | 0.0018267 |
| ESI- | 628.7272876 | 0.0018946 |
| ESI- | 348.0808264 | 0.0018958 |
