# Supplementary material for: Utilizing serum metabolomics for assessing postoperative efficacy and monitoring recurrence in gastric cancer patients
Source: BMC Cancer. 2024 Jan 2;24:27. doi: 10.1186/s12885-023-11786-2 (PMC10763142; doi:10.1186/s12885-023-11786-2)
Supplement: Supplementary file 3 — Supplementary Material 3:The identification of 418 metabolites [file 12885_2023_11786_MOESM3_ESM.docx]

Supplementary table3. The identification of 418 metabolites

| Mass-to-charge ratio | F | P |
| --- | --- | --- |
| 380.1285414 | 86.827 | 1.29E-21 |
| 517.0975415 | 54.23 | 5.87E-17 |
| 815.7605839 | 46.972 | 1.22E-15 |
| 675.2470844 | 46.229 | 1.69E-15 |
| 295.131507 | 43.334 | 6.30E-15 |
| 317.1134044 | 41.256 | 1.68E-14 |
| 983.8290885 | 40.337 | 2.62E-14 |
| 853.517025 | 38.994 | 5.09E-14 |
| 479.2843297 | 38.811 | 5.57E-14 |
| 145.0163565 | 36.958 | 1.43E-13 |
| 587.0079076 | 36.759 | 1.59E-13 |
| 762.0448058 | 36.526 | 1.79E-13 |
| 770.7262352 | 36.503 | 1.81E-13 |
| 982.8278652 | 35.984 | 2.38E-13 |
| 187.1339313 | 35.895 | 2.49E-13 |
| 487.3378319 | 35.716 | 2.74E-13 |
| 282.2783452 | 34.39 | 5.55E-13 |
| 757.3968312 | 34.291 | 5.86E-13 |
| 663.3351846 | 33.915 | 7.19E-13 |
| 967.5819532 | 32.689 | 1.41E-12 |
| 219.0455222 | 32.635 | 1.46E-12 |
| 578.8788816 | 32.478 | 1.59E-12 |
| 554.8997008 | 31.954 | 2.14E-12 |
| 836.6709328 | 31.871 | 2.24E-12 |
| 880.9895855 | 30.996 | 3.69E-12 |
| 443.2710671 | 30.763 | 4.23E-12 |
| 449.201504 | 30.69 | 4.41E-12 |
| 668.315585 | 30.296 | 5.54E-12 |
| 626.9766675 | 30.162 | 5.99E-12 |
| 846.8098981 | 30.124 | 6.13E-12 |
| 441.210079 | 29.763 | 7.58E-12 |
| 576.8819352 | 29.76 | 7.59E-12 |
| 665.2593908 | 29.528 | 8.71E-12 |
| 870.5237867 | 29.047 | 1.16E-11 |
| 890.0867492 | 28.756 | 1.38E-11 |
| 271.1550875 | 28.736 | 1.40E-11 |
| 208.9362444 | 28.677 | 1.45E-11 |
| 709.3418165 | 28.528 | 1.59E-11 |
| 243.114125 | 28.085 | 2.08E-11 |
| 815.6937326 | 28.03 | 2.15E-11 |
| 1166.700845 | 27.884 | 2.35E-11 |
| 473.0708416 | 27.83 | 2.43E-11 |
| 245.139468 | 27.758 | 2.54E-11 |
| 944.4988877 | 27.715 | 2.61E-11 |
| 943.9972726 | 27.567 | 2.85E-11 |
| 270.0561775 | 27.558 | 2.87E-11 |
| 1194.702677 | 27.005 | 4.05E-11 |
| 565.0244005 | 26.992 | 4.08E-11 |
| 725.0629315 | 26.895 | 4.34E-11 |
| 1106.651899 | 26.755 | 4.73E-11 |
| 438.9831485 | 26.732 | 4.80E-11 |
| 460.2082752 | 26.212 | 6.67E-11 |
| 282.2786713 | 25.6 | 9.86E-11 |
| 546.0468957 | 25.329 | 1.17E-10 |
| 980.6004781 | 25.295 | 1.20E-10 |
| 871.1110327 | 25.154 | 1.32E-10 |
| 631.7427877 | 25.089 | 1.37E-10 |
| 930.547663 | 24.98 | 1.47E-10 |
| 272.111143 | 24.908 | 1.54E-10 |
| 460.9651971 | 24.861 | 1.59E-10 |
| 219.1702841 | 24.674 | 1.80E-10 |
| 418.8778366 | 24.409 | 2.14E-10 |
| 945.0003138 | 24.291 | 2.32E-10 |
| 417.0010872 | 24.07 | 2.69E-10 |
| 761.0410084 | 23.926 | 2.96E-10 |
| 937.581432 | 23.757 | 3.31E-10 |
| 188.1372673 | 23.707 | 3.42E-10 |
| 738.4239211 | 23.706 | 3.42E-10 |
| 423.9443855 | 23.666 | 3.52E-10 |
| 724.3836627 | 23.576 | 3.74E-10 |
| 963.6086763 | 23.148 | 4.99E-10 |
| 129.0383968 | 23.006 | 5.50E-10 |
| 722.059916 | 22.906 | 5.89E-10 |
| 819.7876443 | 22.503 | 7.77E-10 |
| 425.9411928 | 22.24 | 9.32E-10 |
| 716.4337901 | 22.201 | 9.57E-10 |
| 780.8054129 | 22.199 | 9.59E-10 |
| 111.0085109 | 22.195 | 9.61E-10 |
| 918.6073853 | 21.988 | 1.11E-09 |
| 654.3376976 | 21.925 | 1.16E-09 |
| 710.3929973 | 21.638 | 1.42E-09 |
| 112.8946828 | 21.437 | 1.63E-09 |
| 510.2292082 | 21.42 | 1.65E-09 |
| 141.1281715 | 21.415 | 1.66E-09 |
| 524.0390736 | 21.37 | 1.71E-09 |
| 682.2919406 | 21.32 | 1.78E-09 |
| 377.2285342 | 21.299 | 1.80E-09 |
| 931.591382 | 21.25 | 1.87E-09 |
| 531.3224227 | 21.193 | 1.94E-09 |
| 427.9385207 | 21.133 | 2.03E-09 |
| 598.3575933 | 20.994 | 2.24E-09 |
| 808.4671154 | 20.948 | 2.31E-09 |
| 738.7250624 | 20.83 | 2.52E-09 |
| 733.051547 | 20.815 | 2.55E-09 |
| 861.4543389 | 20.604 | 2.96E-09 |
| 854.4690499 | 20.541 | 3.10E-09 |
| 368.6252644 | 20.396 | 3.45E-09 |
| 736.7262079 | 20.205 | 3.96E-09 |
| 739.0602596 | 20.091 | 4.30E-09 |
| 703.0825565 | 20.039 | 4.47E-09 |
| 962.6065527 | 19.997 | 4.61E-09 |
| 842.7733534 | 19.977 | 4.68E-09 |
| 445.1267624 | 19.536 | 6.47E-09 |
| 848.5114666 | 19.434 | 6.98E-09 |
| 651.3976837 | 19.384 | 7.24E-09 |
| 740.4297011 | 19.256 | 7.97E-09 |
| 463.2177709 | 19.163 | 8.54E-09 |
| 145.0616326 | 19.107 | 8.90E-09 |
| 777.0161563 | 19.081 | 9.08E-09 |
| 759.3628711 | 19.002 | 9.63E-09 |
| 307.9237434 | 18.997 | 9.66E-09 |
| 376.2252092 | 18.992 | 9.70E-09 |
| 446.2520453 | 18.973 | 9.85E-09 |
| 945.5026095 | 18.842 | 1.09E-08 |
| 755.0337351 | 18.829 | 1.10E-08 |
| 516.3301468 | 18.826 | 1.10E-08 |
| 859.0195608 | 18.807 | 1.12E-08 |
| 1428.747583 | 18.782 | 1.14E-08 |
| 1091.302646 | 18.704 | 1.21E-08 |
| 811.2761162 | 18.439 | 1.47E-08 |
| 740.0632553 | 18.423 | 1.49E-08 |
| 738.9481312 | 18.41 | 1.51E-08 |
| 1473.759096 | 18.343 | 1.59E-08 |
| 602.5661659 | 18.28 | 1.66E-08 |
| 371.9045924 | 18.271 | 1.67E-08 |
| 116.8894554 | 18.253 | 1.70E-08 |
| 783.0242575 | 18.25 | 1.70E-08 |
| 645.2686642 | 18.084 | 1.93E-08 |
| 646.7102642 | 18.045 | 1.99E-08 |
| 917.6047802 | 17.931 | 2.17E-08 |
| 807.9651686 | 17.889 | 2.25E-08 |
| 461.2019173 | 17.855 | 2.31E-08 |
| 263.1039434 | 17.842 | 2.33E-08 |
| 651.3583457 | 17.726 | 2.55E-08 |
| 369.9072839 | 17.699 | 2.60E-08 |
| 522.0414998 | 17.675 | 2.65E-08 |
| 417.2118315 | 17.661 | 2.68E-08 |
| 173.994541 | 17.621 | 2.76E-08 |
| 321.061912 | 17.593 | 2.83E-08 |
| 716.8520311 | 17.466 | 3.12E-08 |
| 127.0510782 | 17.403 | 3.28E-08 |
| 571.0320128 | 17.378 | 3.34E-08 |
| 418.2147242 | 17.322 | 3.49E-08 |
| 611.005061 | 17.187 | 3.88E-08 |
| 415.1477647 | 17.121 | 4.09E-08 |
| 509.3101327 | 17.102 | 4.15E-08 |
| 395.0016668 | 17.076 | 4.24E-08 |
| 754.2563787 | 16.957 | 4.65E-08 |
| 970.0089299 | 16.939 | 4.72E-08 |
| 860.1172304 | 16.905 | 4.85E-08 |
| 805.3457704 | 16.857 | 5.04E-08 |
| 321.9038247 | 16.831 | 5.14E-08 |
| 585.3576577 | 16.657 | 5.90E-08 |
| 376.6236402 | 16.613 | 6.11E-08 |
| 1450.755485 | 16.586 | 6.25E-08 |
| 264.1070964 | 16.46 | 6.91E-08 |
| 959.0776713 | 16.398 | 7.26E-08 |
| 791.760528 | 16.384 | 7.34E-08 |
| 517.3336732 | 16.297 | 7.88E-08 |
| 1239.734718 | 16.242 | 8.23E-08 |
| 722.7506948 | 16.239 | 8.25E-08 |
| 858.9768341 | 16.222 | 8.37E-08 |
| 284.7746255 | 16.177 | 8.68E-08 |
| 694.9223372 | 16.122 | 9.07E-08 |
| 1091.921926 | 16.081 | 9.38E-08 |
| 824.41253 | 16.072 | 9.45E-08 |
| 540.8202198 | 16.05 | 9.62E-08 |
| 953.1748561 | 16.046 | 9.65E-08 |
| 717.3532354 | 16.009 | 9.94E-08 |
| 498.9383469 | 16.005 | 9.98E-08 |
| 392.8910273 | 15.976 | 1.02E-07 |
| 853.9680475 | 15.951 | 1.04E-07 |
| 512.9147128 | 15.855 | 1.13E-07 |
| 444.2120691 | 15.829 | 1.15E-07 |
| 407.1715387 | 15.783 | 1.19E-07 |
| 514.1305494 | 15.775 | 1.20E-07 |
| 264.1070778 | 15.752 | 1.23E-07 |
| 1087.558185 | 15.695 | 1.28E-07 |
| 1091.178316 | 15.683 | 1.30E-07 |
| 774.2233471 | 15.624 | 1.36E-07 |
| 699.4075865 | 15.622 | 1.36E-07 |
| 255.1215167 | 15.613 | 1.37E-07 |
| 398.2552178 | 15.466 | 1.55E-07 |
| 597.8161869 | 15.363 | 1.69E-07 |
| 862.3952328 | 15.304 | 1.77E-07 |
| 393.060644 | 15.205 | 1.92E-07 |
| 462.9620789 | 15.135 | 2.04E-07 |
| 363.0275362 | 15.004 | 2.27E-07 |
| 294.8030875 | 14.985 | 2.31E-07 |
| 474.8668976 | 14.64 | 3.08E-07 |
| 572.8166562 | 14.6 | 3.19E-07 |
| 855.6589832 | 14.569 | 3.27E-07 |
| 389.049857 | 14.47 | 3.55E-07 |
| 954.7653202 | 14.457 | 3.59E-07 |
| 474.9420313 | 14.396 | 3.79E-07 |
| 361.0475452 | 14.312 | 4.07E-07 |
| 649.8026892 | 14.155 | 4.65E-07 |
| 504.3345878 | 14.148 | 4.67E-07 |
| 510.7309705 | 14.124 | 4.77E-07 |
| 648.8180211 | 14.071 | 4.99E-07 |
| 1308.189999 | 14.058 | 5.05E-07 |
| 1053.132655 | 14.044 | 5.11E-07 |
| 744.5618597 | 14.04 | 5.13E-07 |
| 1451.757917 | 14.019 | 5.22E-07 |
| 994.1188454 | 13.92 | 5.68E-07 |
| 1474.761351 | 13.812 | 6.23E-07 |
| 460.0603056 | 13.783 | 6.39E-07 |
| 405.91547 | 13.78 | 6.41E-07 |
| 1480.269643 | 13.774 | 6.44E-07 |
| 778.7126253 | 13.734 | 6.67E-07 |
| 372.9011105 | 13.7 | 6.87E-07 |
| 1391.205541 | 13.665 | 7.08E-07 |
| 669.3071578 | 13.633 | 7.27E-07 |
| 618.2375536 | 13.568 | 7.70E-07 |
| 1264.224887 | 13.53 | 7.96E-07 |
| 638.8131206 | 13.494 | 8.21E-07 |
| 634.8889165 | 13.461 | 8.45E-07 |
| 1090.920515 | 13.42 | 8.75E-07 |
| 348.0808264 | 13.403 | 8.89E-07 |
| 810.4445313 | 13.382 | 9.05E-07 |
| 1457.14531 | 13.364 | 9.19E-07 |
| 443.1814412 | 13.344 | 9.35E-07 |
| 438.0776193 | 13.278 | 9.91E-07 |
| 448.1086591 | 13.213 | 1.05E-06 |
| 620.8741449 | 13.211 | 1.05E-06 |
| 527.2138706 | 13.129 | 1.13E-06 |
| 491.1231739 | 13.114 | 1.14E-06 |
| 341.9992991 | 13.096 | 1.16E-06 |
| 482.0935543 | 13.065 | 1.19E-06 |
| 661.2523035 | 13.065 | 1.20E-06 |
| 916.2285128 | 13.038 | 1.22E-06 |
| 618.2378507 | 12.999 | 1.27E-06 |
| 695.7951419 | 12.981 | 1.29E-06 |
| 1237.483264 | 12.885 | 1.40E-06 |
| 508.1136447 | 12.883 | 1.40E-06 |
| 780.7094594 | 12.826 | 1.48E-06 |
| 658.0662241 | 12.821 | 1.48E-06 |
| 728.8108871 | 12.821 | 1.48E-06 |
| 273.9597533 | 12.755 | 1.57E-06 |
| 817.1455331 | 12.662 | 1.71E-06 |
| 851.1614109 | 12.617 | 1.78E-06 |
| 435.8804597 | 12.482 | 2.01E-06 |
| 477.1021659 | 12.465 | 2.03E-06 |
| 1083.144134 | 12.428 | 2.10E-06 |
| 827.0010669 | 12.415 | 2.13E-06 |
| 586.3614314 | 12.4 | 2.16E-06 |
| 1233.780863 | 12.398 | 2.16E-06 |
| 971.3637948 | 12.36 | 2.24E-06 |
| 292.0868397 | 12.3 | 2.36E-06 |
| 513.3183345 | 12.232 | 2.51E-06 |
| 675.044284 | 12.23 | 2.51E-06 |
| 1427.752287 | 12.196 | 2.59E-06 |
| 644.8490276 | 12.142 | 2.72E-06 |
| 419.8957956 | 12.141 | 2.72E-06 |
| 532.9009768 | 12.071 | 2.90E-06 |
| 369.0082893 | 12.056 | 2.94E-06 |
| 372.1760189 | 12.028 | 3.02E-06 |
| 865.4016689 | 12.013 | 3.06E-06 |
| 1277.262386 | 12.011 | 3.07E-06 |
| 1317.217314 | 11.934 | 3.29E-06 |
| 801.3866271 | 11.93 | 3.30E-06 |
| 552.9803889 | 11.907 | 3.37E-06 |
| 693.2488911 | 11.842 | 3.57E-06 |
| 471.9807647 | 11.832 | 3.61E-06 |
| 1075.112668 | 11.822 | 3.64E-06 |
| 629.8848541 | 11.819 | 3.65E-06 |
| 1088.257302 | 11.812 | 3.67E-06 |
| 950.1560143 | 11.795 | 3.73E-06 |
| 516.3303522 | 11.737 | 3.93E-06 |
| 486.0654031 | 11.663 | 4.21E-06 |
| 1192.635818 | 11.658 | 4.23E-06 |
| 439.2198887 | 11.655 | 4.24E-06 |
| 1045.108681 | 11.652 | 4.25E-06 |
| 716.9349885 | 11.616 | 4.39E-06 |
| 944.2147552 | 11.613 | 4.41E-06 |
| 444.1850279 | 11.549 | 4.68E-06 |
| 1271.746101 | 11.535 | 4.73E-06 |
| 408.9322768 | 11.519 | 4.80E-06 |
| 1474.258482 | 11.492 | 4.92E-06 |
| 886.3826687 | 11.482 | 4.97E-06 |
| 605.1547569 | 11.433 | 5.20E-06 |
| 592.8351266 | 11.422 | 5.26E-06 |
| 638.0934766 | 11.419 | 5.27E-06 |
| 1033.1717 | 11.27 | 6.05E-06 |
| 845.0604786 | 11.263 | 6.09E-06 |
| 741.0826951 | 11.195 | 6.49E-06 |
| 555.8694294 | 11.175 | 6.60E-06 |
| 666.0947721 | 11.115 | 6.99E-06 |
| 629.2280406 | 11.077 | 7.24E-06 |
| 652.2204891 | 11.073 | 7.26E-06 |
| 558.9962243 | 11.039 | 7.50E-06 |
| 704.239406 | 11.023 | 7.61E-06 |
| 881.9900999 | 10.994 | 7.82E-06 |
| 655.7808499 | 10.987 | 7.87E-06 |
| 1274.42997 | 10.973 | 7.97E-06 |
| 697.2707822 | 10.898 | 8.55E-06 |
| 1354.171998 | 10.833 | 9.10E-06 |
| 1315.216418 | 10.816 | 9.24E-06 |
| 598.8527959 | 10.791 | 9.46E-06 |
| 672.2431498 | 10.774 | 9.61E-06 |
| 1061.16267 | 10.713 | 1.02E-05 |
| 708.644113 | 10.616 | 1.12E-05 |
| 481.0920427 | 10.557 | 1.18E-05 |
| 926.8511654 | 10.521 | 1.22E-05 |
| 217.951195 | 10.516 | 1.23E-05 |
| 630.1539011 | 10.511 | 1.23E-05 |
| 628.7272876 | 10.509 | 1.24E-05 |
| 548.1950099 | 10.478 | 1.27E-05 |
| 1429.255391 | 10.472 | 1.28E-05 |
| 893.1385977 | 10.463 | 1.29E-05 |
| 695.9249826 | 10.432 | 1.33E-05 |
| 729.2684427 | 10.377 | 1.40E-05 |
| 704.1318698 | 10.365 | 1.42E-05 |
| 942.2121383 | 10.336 | 1.46E-05 |
| 293.0983193 | 10.28 | 1.54E-05 |
| 696.2626722 | 10.225 | 1.62E-05 |
| 368.9712519 | 10.205 | 1.65E-05 |
| 641.2154505 | 10.187 | 1.68E-05 |
| 673.245612 | 10.04 | 1.93E-05 |
| 928.7476463 | 9.9858 | 2.04E-05 |
| 630.230025 | 9.9786 | 2.05E-05 |
| 628.7272546 | 9.9258 | 2.16E-05 |
| 729.3753232 | 9.9147 | 2.18E-05 |
| 649.8535626 | 9.9105 | 2.19E-05 |
| 661.7538089 | 9.8638 | 2.29E-05 |
| 648.4511723 | 9.8067 | 2.42E-05 |
| 723.2263068 | 9.7674 | 2.52E-05 |
| 822.694587 | 9.7555 | 2.55E-05 |
| 1071.443807 | 9.7327 | 2.60E-05 |
| 699.2686542 | 9.7269 | 2.62E-05 |
| 651.1226646 | 9.6964 | 2.70E-05 |
| 681.0843981 | 9.5797 | 3.02E-05 |
| 441.4223124 | 9.5755 | 3.03E-05 |
| 1451.256989 | 9.5565 | 3.09E-05 |
| 319.2616298 | 9.5389 | 3.15E-05 |
| 660.8576919 | 9.4932 | 3.29E-05 |
| 1066.629203 | 9.4759 | 3.35E-05 |
| 655.2236088 | 9.434 | 3.49E-05 |
| 1233.298666 | 9.4338 | 3.49E-05 |
| 682.3610126 | 9.4229 | 3.52E-05 |
| 428.9711536 | 9.4042 | 3.59E-05 |
| 502.8580098 | 9.4001 | 3.60E-05 |
| 680.2279195 | 9.3719 | 3.70E-05 |
| 1099.116574 | 9.3233 | 3.89E-05 |
| 719.2508985 | 9.2724 | 4.09E-05 |
| 760.9615185 | 9.26 | 4.14E-05 |
| 991.3421449 | 9.2593 | 4.14E-05 |
| 1153.703822 | 9.2043 | 4.37E-05 |
| 408.8641953 | 9.158 | 4.58E-05 |
| 680.7510502 | 9.047 | 5.11E-05 |
| 629.1511758 | 8.9842 | 5.44E-05 |
| 707.4952391 | 8.9701 | 5.51E-05 |
| 399.0619116 | 8.9597 | 5.57E-05 |
| 524.2264462 | 8.9237 | 5.77E-05 |
| 1452.261464 | 8.8998 | 5.91E-05 |
| 662.4666206 | 8.8765 | 6.05E-05 |
| 189.0959334 | 8.8411 | 6.27E-05 |
| 710.2740142 | 8.7472 | 6.89E-05 |
| 981.2382584 | 8.731 | 7.00E-05 |
| 451.1083783 | 8.6888 | 7.30E-05 |
| 605.3199146 | 8.6193 | 7.83E-05 |
| 469.2915145 | 8.6058 | 7.94E-05 |
| 470.4562872 | 8.5196 | 8.66E-05 |
| 578.8684713 | 8.4688 | 9.12E-05 |
| 686.2531878 | 8.4049 | 9.73E-05 |
| 722.2695923 | 8.3583 | 0.00010197 |
| 964.32641 | 8.3021 | 0.00010796 |
| 1062.153443 | 8.2051 | 0.00011917 |
| 656.3171663 | 8.1728 | 0.00012317 |
| 457.2427345 | 8.1058 | 0.00013189 |
| 449.3440607 | 8.0346 | 0.00014186 |
| 741.2931364 | 7.9928 | 0.00014808 |
| 696.3754671 | 7.8646 | 0.00016896 |
| 350.1502391 | 7.7177 | 0.00019665 |
| 942.3444227 | 7.5964 | 0.00022303 |
| 1193.639312 | 7.5654 | 0.00023033 |
| 917.8393336 | 7.4736 | 0.00025346 |
| 697.2704273 | 7.4281 | 0.00026579 |
| 156.0488752 | 7.3503 | 0.00028833 |
| 402.2368654 | 7.2472 | 0.00032126 |
| 793.4378169 | 7.0412 | 0.00039922 |
| 618.1787267 | 6.9669 | 0.00043191 |
| 411.1665539 | 6.9541 | 0.00043783 |
| 673.3075029 | 6.9207 | 0.00045362 |
| 664.7579012 | 6.8403 | 0.00049409 |
| 966.3313535 | 6.789 | 0.00052189 |
| 793.9401713 | 6.7808 | 0.00052644 |
| 792.9382063 | 6.7095 | 0.00056813 |
| 740.2895834 | 6.5228 | 0.00069402 |
| 1395.548125 | 6.4818 | 0.00072537 |
| 627.5549541 | 6.2909 | 0.00089146 |
| 432.186184 | 6.2892 | 0.00089312 |
| 350.2097076 | 6.2492 | 0.0009327 |
| 485.2813382 | 6.2129 | 0.00097013 |
| 1468.812201 | 6.145 | 0.0010445 |
| 716.7669911 | 6.1308 | 0.0010608 |
| 547.3203928 | 6.0331 | 0.0011801 |
| 1020.500494 | 5.8595 | 0.0014271 |
| 164.8360293 | 5.828 | 0.0014773 |
| 704.767281 | 5.8242 | 0.0014835 |
| 405.3673136 | 5.7122 | 0.0016783 |
| 1149.654532 | 5.6637 | 0.0017706 |
| 1155.848228 | 5.6602 | 0.0017773 |
| 812.3926156 | 5.6081 | 0.0018828 |
| 453.1660051 | 5.5176 | 0.0020815 |
| 638.780563 | 5.4939 | 0.0021369 |
| 580.5576246 | 5.3872 | 0.0024061 |
| 128.0688513 | 5.3834 | 0.0024164 |
| 688.783953 | 5.2224 | 0.0028924 |
| 1108.594639 | 5.0511 | 0.0035058 |
| 1125.654096 | 5.0249 | 0.0036108 |
| 1072.446125 | 5.0069 | 0.0036846 |
| 618.5485171 | 4.994 | 0.0037386 |
| 963.7552667 | 4.6808 | 0.0053304 |
| 545.3393141 | 4.6769 | 0.0053543 |
| 180.9733947 | 4.5789 | 0.0059865 |
| 1198.905299 | 4.4716 | 0.0067676 |
| 552.7371836 | 4.4417 | 0.0070037 |
